# A brief simulation intervention increasing basic science and clinical knowledge

**DOI:** 10.3402/meo.v21.30744

**Published:** 2016-04-07

**Authors:** Maria L. Sheakley, Gregory E. Gilbert, Kim Leighton, Maureen Hall, Diana Callender, David Pederson

**Affiliations:** 1 Department of Biomedical Sciences, Western Michigan University Homer Stryker M.D. School of Medicine, Kalamazoo, MI, USA; 2 Center for Teaching and Learning, Ross University School of Medicine, Commonwealth of Dominica, West Indies; 3 DeVry Medical International's Institute for Research and Clinical Strategy, Iselin, NJ, USA; 4 The Commonwealth Medical College, Scranton, PA, USA

**Keywords:** Manikins, high-fidelity simulation, undergraduate medical education, teaching, cardiovascular course

## Abstract

**Background:**

The United States Medical Licensing Examination (USMLE) is increasing clinical content on the Step 1 exam; thus, inclusion of clinical applications within the basic science curriculum is crucial. Including simulation activities during basic science years bridges the knowledge gap between basic science content and clinical application.

**Purpose:**

To evaluate the effects of a one-off, 1-hour cardiovascular simulation intervention on a summative assessment after adjusting for relevant demographic and academic predictors.

**Methods:**

This study was a non-randomized study using historical controls to evaluate curricular change. The control group received lecture (*n*_l_=515) and the intervention group received lecture plus a simulation exercise (*n*_l+s_=1,066). Assessment included summative exam questions (*n*=4) that were scored as pass/fail (≥75%). USMLE-style assessment questions were identical for both cohorts. Descriptive statistics for variables are presented and odds of passage calculated using logistic regression.

**Results:**

Undergraduate grade point ratio, MCAT-BS, MCAT-PS, age, attendance at an academic review program, and gender were significant predictors of summative exam passage. Students receiving the intervention were significantly more likely to pass the summative exam than students receiving lecture only (*P*=0.0003).

**Discussion:**

Simulation plus lecture increases short-term understanding as tested by a written exam. A longitudinal study is needed to assess the effect of a brief simulation intervention on long-term retention of clinical concepts in a basic science curriculum.

The United States Medical Licensing Examination (USMLE) Step 1 progressively includes more clinical content, such as vignette-style questions and audio–visual clips (1). Therefore, the need to include clinical applications within basic science curricula in medical school is crucial. Furthermore, as technology improves, simulated clinical activities offer novel varieties of experiences for medical students, beginning in year 1. Including more simulation activities during the basic science years may help to bridge the gap in student knowledge between basic science content and clinical skills application. Harvey^®^ The Cardiopulmonary Patient Simulator (2) is a powerful tool when paired with pertinent lecture content, adding variability to the learning experience and aiding transfer of knowledge to clinical practice (3). In addition, there is evidence supporting simulation in the basic science curriculum, as Chakravarthy (4) observed knowledge retention might be increased by presenting as much of the basic science education as possible in a clinical context. Simulation also allows students to develop self-confidence and competence in basic clinical skills and learn about patient safety before being exposed to patients (5). The purpose of this study was to evaluate the effects of a one-off, 1-hour cardiovascular simulation intervention on a summative assessment after adjusting for relevant demographic and academic predictors.

## Literature review

Evidence exists that learning with simulation leads to satisfaction with the learning experience, increased confidence, self-reported increase in knowledge, and ability to perform skills better (6). However, one of the reasons for the lack of evidence for the efficacy of simulation-based medical education in demonstrating higher level outcomes is because simulation is often embedded in the curriculum, leading to challenges when researchers attempt to demonstrate that learning with simulation leads to specific outcomes (7). It is difficult to separate individual teaching strategy from the entirety of methods used by faculty to provide education on a specific topic in order to examine the efficacy of the individual strategy.

Traditionally, undergraduate medical basic science curricula are taught using a lecture format. However, for a variety of reasons, lectures do not foster critical thinking or student engagement, and there is little research to indicate that sustained learning occurs as a result of this teaching strategy (8). In addition, retention of basic science knowledge beyond medical school is a well-documented longstanding challenge (9). Although few studies could be located examining short-term outcomes of simulation in medical schools, there is evidence of simulation leading to significant increases in confidence and short-term knowledge retention. For example, in a study by Vadnais et al. (10), physicians showed improved comfort and objective measures of knowledge immediately after taking part in a simulation activity.

Adding active learning strategies, such as simulation, to the lecture format of basic science education has been shown to positively impact exam scores in the short term (11). The use of deliberate practice strategies providing opportunities to repetitively practice skills leads to increased retention (6) but may be time-consuming to add to traditional lecture curricula. The addition of simulation in medical school education has been shown to increase knowledge and self-efficacy for communication and safety skills (5) and empathy (12). Outcomes of simulation in graduate medical education are better documented (4), leading to numerous opportunities to establish effectiveness of simulation in the first 2 years of medical education.

## Methods and materials

This study was a non-randomized educational study using historical controls as a means for evaluation of a curricular change. This investigation was approved by the Institutional Review Board of a large offshore US medical school where the study was conducted and subscribed to the tenets of the Declaration of Helsinki. Informed consent to participate in the medical simulation research study was accomplished by voluntary completion and return of a survey questionnaire.

### Sample

Two groups of students participated in this investigation. The first group was a historical control of 515 students from the 2009 matriculating class at the medical school. This was the most recent group of students receiving the traditional curriculum (lecture only). The group receiving the intervention (lecture plus simulation) was a group of 1,066 students from the 2011 matriculating class at the same medical school.

### Instruction

For both cohorts, didactic lectures in cardiovascular physiology, gross anatomy, microanatomy, and embryology were presented over a 2-week period, delivered by the same faculty, and covering the same content. Gross anatomy and physiology topics, including ‘gross anatomy of the heart’ (2 hours), ‘cardiac cycle and pressure-volume loops’ (2 hours), and ‘physiological basis of heart sounds and murmurs’ (2 hours), were used as the basis for development of the high-fidelity simulation exercise used in the intervention. Content delivered in these lectures was based on learning objectives provided by the American Physiological Society and American Association of Anatomists.

### Intervention

The intervention group received didactic lectures described in the *Instruction* section and participated in a 1-hour small group simulation exercise designed to reinforce learning objectives of the didactic lectures for cardiac gross anatomy, cardiac cycle, pressure-volume loops, and heart sounds and murmurs. The following are the learning objectives for this simulation. Learning objective 1 is a clinical objective not covered prior to simulation. The underlying concepts covered in objectives 2–5 were discussed in gross anatomy and physiology lectures prior to the simulation exercise, and students performed these objectives for the first time during the simulation.

1. Identify the parts of the stethoscope and demonstrate its proper use and sanitization protocols.
2. Inspect the anterior chest wall for the anatomical location of the apical impulse.
3. Palpate for first heart sound (S1), second heart sound (S2), carotid pulse, and heaves/lifts while verbalizing the anatomically correct areas.
4. Listen for S1 and S2 in all four areas and describe the physiology of S1 and S2 while timing with the carotid pulse and relate the finding to the cardiac cycle.
5. Identify a split S2 upon inspiration over the pulmonic area (valve) and its correlation to the pressure/volume curves.

**Fig. 1:**
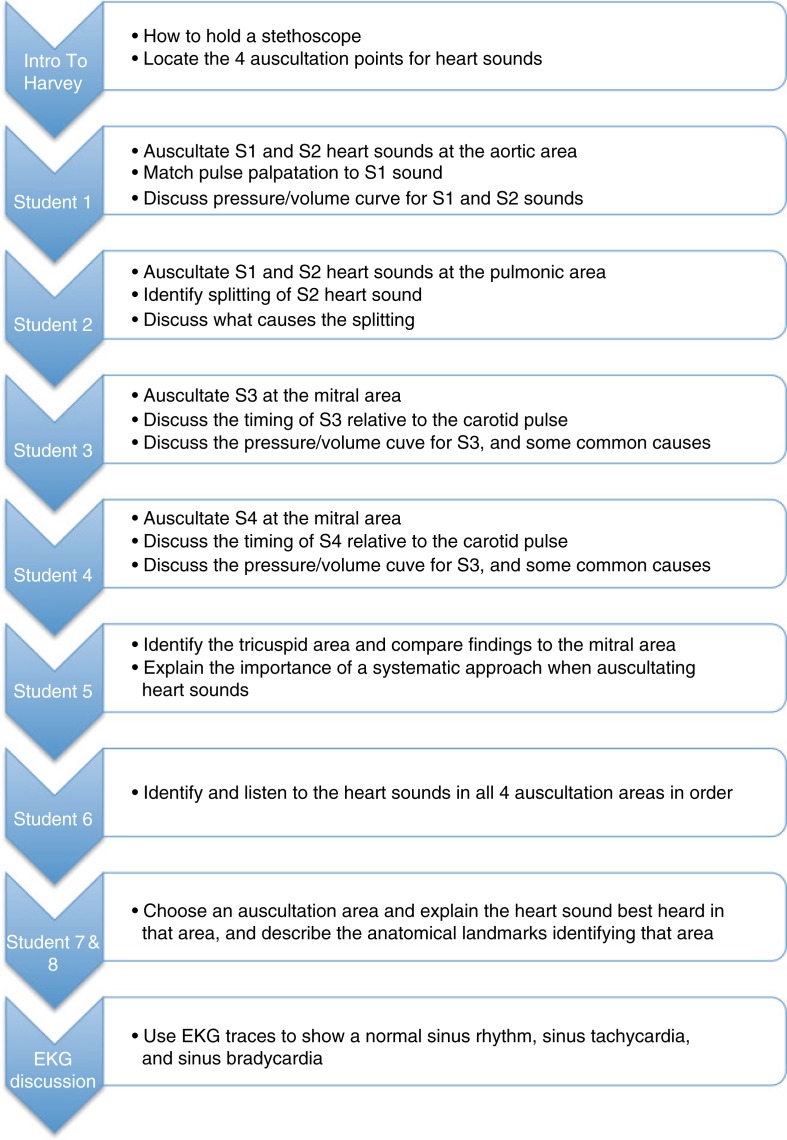
Flow chart for simulation instructors depicting the standardized approach, to ensure uniformity in topics covered and student involvement during the simulation session.

The Harvey^®^ Cardiopulmonary Patient Simulator (2) was used for simulation activities and each group of students completed clinical tasks including proper stethoscope placement for auscultation points on the chest; auscultation of first, second, third, and fourth heart sounds; and auscultation of split heart sounds, with and without respiratory changes. These topics were first described in didactic lectures, then applied during the simulation exercise. Each student had an opportunity to interact directly with the simulator during the session while others observed and listened to the heart sounds and murmurs using the sound transmission system. The simulation protocol detailing progression through the simulation scenario is provided in Fig. 1. Students were also allowed to practice independently over the following weeks.

Clinically trained simulation facilitators viewed cardiovascular lectures to ensure a common understanding of what was taught. Facilitators were given handouts outlining simulation objectives, content, and desired instructional technique. A simulation training session was developed where facilitators discussed objectives and content, and a standardized approach to facilitation was demonstrated. The standardized approach can be viewed in Fig. 1. Facilitators were debriefed following each set of simulations, and videos of the simulations were reviewed with facilitators to improve the process and consistency among facilitators. This iterative process was repeated after each cohort completed the simulation.

### Independent variables

The independent variable assessed in this investigation was the curriculum type, specifically a lecture only versus lecture plus simulation curriculum.

### Dependent variables

Assessment of the cardiovascular course learning outcomes occurred via a summative exam administered on the final day of the course. Assessment questions were higher level Bloom's taxonomy (apply and analyze), written in USMLE style, and identical for both cohorts. There were four assessment questions on the summative exam, all directly mapped to shared learning objectives for didactic lectures and simulation exercise (see Appendix A). Scores were entered as percent correct. Assessments were used to track differences in performance between the intervention group and historical control group. Scores reported in Table 1 reflect performance only on specific questions related to the lectures/simulation activity (four summative exam questions). Validity and reliability of the questions (and instruments) has not been assessed. Tests were administered through and data collected using Questionmark^®^ software for delivering assessments (13).

**Table 1:** Means (standard errors) and relative frequencies (frequencies) for students in the control cohort (lecture) and intervention cohort (lecture+simulation)

Variable	Lecture	Lecture+simulation	*P*
Sample size	515	1,066	
Age	25 (0.18)	25 (0.11)	0.0004
Female	49% (250)	48% (513)	0.9144
Race			
Asian	36% (132)	32% (241)	0.3865
Black non-Hispanic	12% (43)	15% (110)	
White non-Hispanic	39% (141)	38% (282)	
Other	14% (50)	15% (113)	
MERP	23% (120)	29% (313)	0.0114
uGPR	3.2 (0.02)	3.1 (0.01)	0.0159
uGPR (Pre-Req)	3.1 (0.02)	3.0 (0.01)	0.5153
MCAT-BS	8 (0.07)	8 (0.07)	0.8337
MCAT-PS	8 (0.07)	8 (0.06)	0.5525
MCAT VB	8 (0.09)	7 (0.07)	0.7307
MCAT WR			
J	0.4% (2)	0.1% (1)	0.4593
K	1.4% (7)	2.1% (22)	
L	5.9% (30)	7.9% (82)	
M	22.9% (117)	24.6% (256)	
N	9.8% (50)	10.2% (106)	
O	14.5% (74)	12.5% (130)	
P	10.5 (54)	11.2% (117)	
Q	21.9% (112)	20.1% (209)	
R	9.2% (47)	9.0% (94)	
S	3.7% (19)	2.4% (25)	
Summative question performance	66.2 (1.19)	71.5 (0.75)	0.0006
Summative question passage	57.4% (286)	66.8% (701)	0.0004

### Control variables

Many demographic and academic variables are related to medical school performance. In this investigation, we used regression analysis to control for participant differences. Age at matriculation, self-identified gender, and race are known to influence academic performance in medical school and were included in preliminary models. Candidate academic control variables were overall undergraduate grade point ratio (uGPR), undergraduate grade point ratio of pre-requisite courses (uGPR-Pre), and individual MCAT scores (biological sciences, physical sciences, verbal, and writing scores). A final variable we felt important to control for was whether students attended a university-sponsored medical school preparatory program. The Medical Education Review Program (MERP) is a 15-week program offered to students granted conditional acceptance to the medical school and provides students with additional academic preparation prior to medical school matriculation. For a more complete explanation of MERP, see the article by Lindner et al. (14).

### Analysis

Data were entered into a Microsoft Excel^®^ (15) spreadsheet and saved in a comma-separated value (CSV) file. Means and standard errors were calculated for the lecture group (control) and the lecture plus simulation group (intervention) for quantitative variables. For categorical variables, percentages are presented. The Wilcoxon rank sum (Mann–Whitney U) test was used to test if quantitative variables differed significantly and a chi-square test was used to test for a significant difference in categorical variables. Because of the extremely limited range, summative exam scores for the four pre-identified questions were converted to pass/fail using a criterion of ≥75% as passing. Logistic regression was used to ascertain the odds of passage of the exam, independently, after adjustment for covariates. A model building approach using likelihood ratio testing was used to determine if variables contributed significantly to the model. A *P*-value of 0.2 was used as criteria for inclusion in the regression model. Candidate admission variables were entered into models and likelihood ratio testing was used to determine the most parsimonious models. To assess MERP as a mediating factor, a subanalysis was conducted. As this was an educational intervention, an a priori alpha level of 0.10 was specified (16). Results from the final models are presented. All analyses were done using R software (17).

## Results

Descriptive results were calculated for the two groups and are presented in Table 1. Means and standard errors are presented for continuous variables and percentages and relative frequencies are presented for categorical variables.

### Univariate results

Analysis showed statistical differences between the cohorts for three variables. Students in the intervention group were statistically significantly (*P*=0.0004) older than students in the control group, although this age difference was of no practical significance. A significantly greater (*P*=0.0114) proportion of students in the intervention group attended MERP (29% vs. 23%, respectively). In addition, students in the intervention group had a significantly lower uGPR (*P*=0.0159). Scores for the specified questions on the summative exam, and passing rates on those questions, were significantly higher for students in the intervention group than in the control group (*P*=0.0006 and 0.0004, respectively). Furthermore, these results were of practical importance. Students in the intervention group had a mean passing score on the related questions of 71.5% versus the control group average of 66.2%, an almost 6% point increase. In addition, there was an increased statistically significant pass rate of about 10% (*P*=0.0004).

### Summative course exam

uGPR, MCAT Biological and Physical Science scores, matriculation age, whether a student attended MERP or not, and gender (reference: male) were all statistically significant predictors of summative exam passage. Students receiving simulation training in addition to lecture were significantly more likely to pass the summative course exam than students in the lecture only group (*P*=0.0003). Students receiving simulation training were 1.5 times (95% CI: 1.2, 1.9) as likely to pass the summative exam as students receiving just lecture, effect size 0.23 (95% CI: −5.14, 4.68; *P*=0.93).

## Discussion

Students often have difficulty understanding and integrating basic science content with clinically relevant topics. In a study to determine if medical students’ perceived relevance of whether biomedical science would impact retention of basic science knowledge, Malau-Aduli et al. (18) found teaching strategies that increase awareness of clinical relevance improves retention of knowledge. The development of this brief, high-fidelity cardiovascular simulation exercise in direct conjunction with basic science lecture content was intended to bridge the gap between basic science and clinical knowledge through the use of clinical application, as assessed by clinical vignette-style multiple choice questions in a written exam.

Since this was a non-randomized study using historical controls, analysis was done to compare the two cohorts to account for significant differences in quantitative and categorical variables. The analysis showed a significant difference between the two cohorts for three variables: matriculation age, MERP attendance, and uGPR. The intervention group was statistically older than the control group; however, there was no practical difference in age. The intervention group also has a significantly higher proportion of students who attended and successfully completed MERP prior to matriculation. This could have benefited the cohort, since the students attending MERP were exposed to cardiovascular anatomy and physiology lectures during MERP; nevertheless, the simulation experience was novel to these students as well. On the contrary, students attending MERP had lower uGPR and MCAT (physical science, biological science, and verbal) than non-MERP students, possibly disadvantaging them academically. However, a subanalysis of students not participating in MERP showed no change in the odds of passing the summative examination.

Students in the intervention group had a statistically lower uGPR. This is not surprising since the intervention group had a higher number of students participating in MERP and had on average lower uGPR and MCAT scores. What is surprising, however, is that there was no significant difference in undergraduate prerequisite grade point ratio between the two groups. Through this we might imply students performed equally in the prerequisite sciences; however, they did not perform equally in their other coursework. This grade point ratio differential could be the result of a number of underlying causes, including time management capabilities, that is, students spending more time studying for prerequisite courses than other courses, or poor performance in classes taken earlier in a student's college career.

Students participating in a high-fidelity cardiovascular simulation exercise following a series of basic science cardiovascular lectures performed significantly better on the related questions on the cardiovascular summative exam than those who received lectures only after controlling for uGPR, MCAT biological and physical science scores, matriculation age, MERP attendance, and gender. This exam was administered on the last day of the cardiovascular course, within 1 week of completing the lectures and simulation. Pairing simulation with cardiovascular physiology lectures allowed students opportunities to apply basic science knowledge to a clinical scenario, thereby enhancing the ability to assimilate and contextualize knowledge better, which was demonstrated by enhanced performance of the intervention group on the relevant questions in the summative course exam.

On course evaluation, 54% of students strongly agreed and 40% of students agreed that the simulation session was a ‘valuable and rewarding learning experience’, as is echoed in the literature (19). Future research in this area should include randomized controls and greater exposure to simulation, since greater exposure to simulation in the curriculum may further enhance retention by presenting basic science content in a clinically relevant framework, according to Harris et al. (20). Further, more longitudinal data points would help to see if retention of knowledge occurs. Test items and cumulative testing to keep students more continuously engaged in content is needed to more comprehensively examine the effects of simulation on knowledge and knowledge retention when associated with basic science courses.

## Conclusions

Students receiving lecture plus simulation were significantly more likely (1.5 times) to achieve a passing score on exam questions related to cardiac learning events on the summative course exam, compared with those receiving lecture only after controlling for uGPR, MCAT biological sciences and physical science score, MERP attendance, matriculation age, and gender. A longitudinal study is needed to detect differences in clinical skills performance in clinical years. This investigation indicates that simulation in addition to lecture increased short-term knowledge retention and understanding as tested by a written exam.

## Appendix A

Assessment questions used in the summative exam

1) During a routine physical exam, a physician detects a diastolic murmur. To further examine this finding, the following data are collected via cardiac catheterization.

Right atrial diastolic pressure=12 mmHg
Right ventricular pressure=15/0 mmHg
Left atrial pressure=3 mmHg
Left ventricular pressure=100/0 mmHg

Based on the above findings, which of the following is the most likely diagnosis?

- Mitral valve stenosis
- Aortic valve stenosis
- Tricuspid valve stenosis*
- Mitral valve regurgitation
- Aortic valve regurgitation

2) A 50-year-old man visits his physician for an annual exam. He complains of mild breathlessness upon exertion. Upon cardiac auscultation, the physician detects an S3 heart sound. During which phase of the cardiac cycle does the S3 heart sound occur?

- Atrial contraction
- Isovolumetric contraction
- Rapid ejection
- Slow ejection
- Isovolumetric relaxation
- Rapid filling*
- Slow filling

3) The image below shows a frontal view of the heart. Stenosis of the heart valve labeled “X” would produce what abnormal heart sound and murmur?

- S3 heart sound and a systolic murmur
- S3 heart sound and a diastolic murmur
- S4 heart sound and a systolic murmur*
- S4 heart sound and a diastolic murmur

4) You are examining a patient by auscultating his heart while palpating his carotid pulse. If you hear a heart sound just before the carotid pulse is felt (during the atrial contraction phase of the cardiac cycle), which heart sound is it?

- S1
- S2
- S3
- S4*
- Split S1
- Split S2
